# Serum Protein Electrophoresis Bands As Biomarkers for Drug-Sensitive Pulmonary Tuberculosis

**DOI:** 10.7759/cureus.44424

**Published:** 2023-08-31

**Authors:** Poonam Sinha, Ranjay K Ranjan, Manish Shankar, Archana Bharti, Ravi Shekhar

**Affiliations:** 1 Biochemistry, Indira Gandhi Institute of Medical Sciences, Patna, IND; 2 Forensic Medicine and Toxicology, Nalanda Medical College and Hospital, Patna, IND; 3 Pulmonary Medicine, Indira Gandhi Institute of Medical Sciences, Patna, IND

**Keywords:** ntep guidelines, poverty, antituberculosis drugs, serum protein electrophoresis, tuberculosis

## Abstract

Introduction: India has the highest cases of tuberculosis worldwide. According to WHO (2022), the incidence of tuberculosis in India is 210 per 100,000 population. Their incidence of new positive smear cases is 75 per 100,000 population per year. In tuberculosis, the level of albumin decreases while globulin increases leading to a low albumin to globulin (A/G) ratio, and electrophoresis of serum proteins are good diagnostic approach and provides essential information for monitoring treatment outcomes.

Materials and methods: The present study includes 50 cases of pulmonary tuberculosis and 50 age-sex-matched healthy controls. Initially, serum protein estimation and electrophoresis were performed in newly diagnosed patients and controls. All drugs were given as National Tuberculosis Elimination Programme (NTEP) guidelines and blood samples were collected at two-month, four-month, and six-month intervals, and different serum protein fractions were compared and analyzed.

Results: The total serum protein was significantly lower in the cases than in the controls; 6.12±0.61 vs. 7.02±0.56 g/dL (p˂0.0020, t-value=3.12). The mean serum albumin was also significantly lower in the cases compared to the controls; 1.65±0.69 vs. 3.87±0.47g/dL (p˂0.0001, t-value=10.98). The α1 globulin started to rise after four months of treatment and at six months level was 0.262±0.32 g/dL. The level of γ globulin continuously decreases after antituberculous treatment to 1.56±0.67 gm/dL at six months.

Conclusion: The cause of the decrease in total protein and albumin may be due to malnutrition leading to low cellular immunity. Serum protein level and protein electrophoresis should be analyzed as routine tests in patients before, during, and after treatment. It helps us in identifying patients at risk of pulmonary tuberculosis as well prognosis of the disease. This study is a valuable guide in deciding the effective management of tuberculosis patients with drug treatment plans and appropriate dietary intake. Hence, it highlights the complex relationship that exists between poverty and disease.

## Introduction

India has the highest cases of tuberculosis worldwide. According to WHO (2022), the incidence of tuberculosis in India is 210 per 100,000 population. When this data is compared with the year 2015 (incidence 256/lakh of population in India); there has been a decrease of 18% in tuberculosis patients which is 7% better than the global average of 11% [1]. Every year approximately 10 million population develop tuberculosis and 1.5 million die from the disease. Their incidence of new positive smear cases is 75 per 100,000 population per year. The worldwide target is to estimate at least 70% of total cases [2]. Approximately 5,000 people develop the disease per day and 10-15 people are about to get infected from infectious cases every year with an estimated 22% infection rate [3]. In India, approximately 2.7 million people suffer from tuberculosis and more than 4 lakh people die from this disease. Poverty itself is a risk factor for the development of tuberculosis [4]. This increasing trend of pulmonary tuberculosis (PTB) focused attention on the development of molecular tools and assays for rapid and early diagnosis of *Mycobacterium tuberculosis* infection.

*Mycobacterium tuberculosis* is transmitted by droplet infection. Droplet nuclei, due to their small size, escape the respiratory defense mechanism and reach to terminal alveoli [5]. This causes Ghon complex formation. Lymphatics spread causes the bacilli to enter hilar lymph nodes and from there by hematogenic route spreads the whole body [6]. A positive immune response can stop bacilli multiplication. If the tuberculin skin test is positive, it indicates there is an infection [7]. In some cases, as their immune response is decreased, the bacilli multiply and manifestations of disease occur in a few months. Primary infected patients have no clinical symptoms but a tuberculin skin test is positive [8]. Miliary tuberculosis or tuberculous meningitis may develop in immunocompromised patients. Dormant bacilli may persist for a long period and after reactivation produce secondary tuberculosis. Earlier symptoms and signs are vague. Patients mainly present with anorexia, weight loss, general malaise, weakness, fever, and sweating at night [9]. In a large number of cases, initially, a non-productive cough develops into purulent sputum, with or without blood streaking. Erosion of cavity blood vessels causes massive hemoptysis. Parenchymal lesions cause chest pain, and later on dyspnea and, rarely, adult respiratory distress syndrome (ARDS). Extra-PTB involves the genitourinary tract, lymph nodes, meninges, peritoneum, pericardium, bones, and joints. In HIV-infected individuals, extra-PTB is mostly seen. Either acute or chronic inflammation causes changes in levels of serum proteins. In tuberculosis disease, the level of albumin decreases while globulin increases leading to a low albumin to globulin (A/G) ratio. Albumin has the highest proportion among serum proteins. Low levels of serum albumin indicate poor health and predict bad outcomes [10]. The term globulin is high molecular weight, and electrophoretic migration rates slower than albumin. Electrophoresis of various serum proteins are good diagnostic approach and it provides important information for monitoring treatment outcomes. Protein electrophoresis categorizes globulins into four categories: alpha1, alpha2, beta1, beta2, and gamma globulins [11]. The present study aimed to evaluate and monitor changes in serum protein bands before and after first-line antituberculous treatment (ATT) at regular intervals in drug-sensitive PTB patients.

## Materials and methods

The prospective observational study was performed from October 2019 to August 2020 in the Department of Biochemistry, Indira Gandhi Institute of Medical Sciences (IGIMS), Patna. The present study was approved by the Institutional Ethics Committee, vide letter no. 952/IEC/IGIMS/2019. The patient’s record was kept confidential. The present study includes 50 confirmed cases of PTB and 50 age-sex-matched healthy controls. After obtaining informed consent and institutional ethical approval, the cases were selected from the Department of Pulmonary Medicine, IGIMS, Patna for a year. Blood samples were collected from both newly diagnosed smear-positive patients and controls in plain vials and were allowed to stand for 30 minutes. Blood was also collected at two months, four months, and six months after ATT. The treatment was planned according to the National Tuberculosis Elimination Programme (NTEP) [12]. Clotted blood was centrifuged and serum was separated. Following biochemical tests were performed in both patients and controls.

1. Serum proteins albumin and globulin estimation

2. Serum protein electrophoresis

The inclusion criteria include:

1) Patients belonging to either sex in the age group 18-60 years

2) Newly diagnosed smear-positive cases of PTB

3) Apparently disease free age sex-matched healthy controls

Exclusion criteria include:

1) Relapse, treatment failure, defaulter cases of PTB

2) Patients suffering from liver diseases, malabsorption syndrome

3) Pregnancy and lactation

4) HIV patients

5) Extra-PTB

Total serum protein was estimated by the biuret test. Albumin was estimated by the bromocresol green method. Total protein and albumin were estimated after running calibration and quality controls. Total serum protein is composed of albumin and globulins. The normal level of total protein is 6.3-8.2 g/dL; albumin is 3.5-5.5 g/dL; α-1 globulin is 0.24-0.5 g/dL; α-2 globulin is 0.5-1.1 g/dL; β globulin is 0.58-1.20 g/dL; and γ-globulin is 0.6-1.5 g/dL [13].

Serum protein electrophoresis

According to the manufacturer's instructions, electrophoresis was performed on agarose gel by automated Interlab Pretty (Interlab S.R.L., Rome, Italy). A total of 30 μl serum was applied on the sample loader for each sample. Programming for serum protein electrophoresis was done. After completion of electrophoresis gels were dried, and placed on a flat-bed scanner for densitometric analysis. Results were displayed in Elfolab software (Interlab S.R.L., Rome, Italy). Protein fractions were analyzed on the resulting electrophoretogram and visually on the gel. The relative distance is calculated by measurement of each point from the mid-point (in mm) to the mid-point of the albumin peak (in mm). Fractions are named as follows: the first anodal peak was albumin after that α- and β-globulins were placed; gamma-globulins were almost some distance from the point of application. α-globulins further subdivided into α1 and α2 fraction; β-globulins into β1 and β2 fraction.

Statistical analysis

The data analysis was performed by GraphPad Prism software 6.01 version (GraphPad Software, Inc., USA). The numerical data for each analyte was calculated in terms of mean±SD. Comparison between the two groups was performed by unpaired "t-test". The p-value of <0.05 was considered statistically significant.

## Results

The study comprises 50 cases and 50 controls. The female predominance (36%) was less in the tuberculosis group than in the controls (40%). The mean ages in cases and controls were 38.91±12.87 vs. 37.93±12.73 respectively. There is no significant difference between cases and controls (p=0.433) (Table 1).

**Table 1:** Demographic distribution of the study population

Gender	Cases	Controls	p-value
Male	32	30	0.433
Female	18	20	
Mean age (years)	38.91±12.87	37.93±12.73	
Total	50	50	

**Figure 1:**
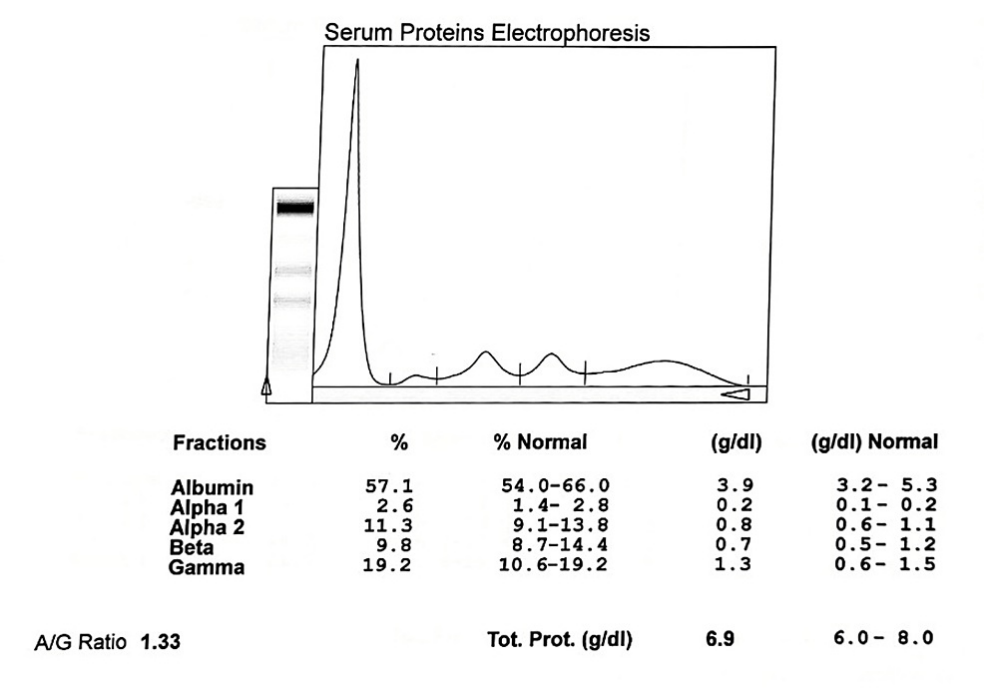
Electrophoretogram of normal healthy controls A/G: albumin to globulin ratio

**Figure 2:**
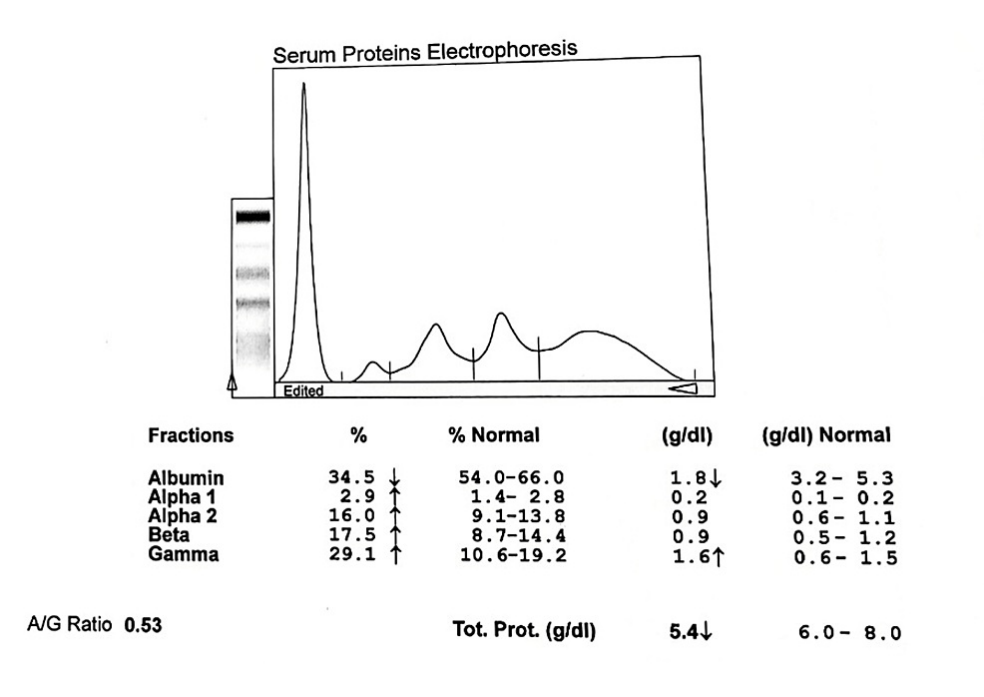
Electrophoretogram of newly diagnosed smear-positive PTB cases A/G: albumin to globulin ratio, PTB: pulmonary tuberculosis

The serum biochemical analysis in newly diagnosed smear-positive cases and controls showed significant differences in levels of total protein, albumin, α1 globulin, and γ globulin. The total serum protein was significantly lower in the newly diagnosed cases than in the controls; 6.12±0.61 vs. 7.02±0.56 g/dL (p˂0.0020, t-value=3.12) respectively. Similarly, the mean serum albumin was also significantly lower in the newly diagnosed cases compared to the controls; 1.65±0.69 vs. 3.87±0.47g/dL (p˂0.0001, t-value=10.98) respectively. Hypoalbuminemia was significantly higher in cases than in controls. Similarly, hypergammaglobulinaemia is prevalent in the cases compared to the controls as γ globulin in cases and controls are 2.70±0.27 and 1.12±0.21 g/dL (p˂0.0001, t-value=12.56) respectively. The α1 globulin is significantly decreased in cases and controls as 0.198±0.04 and 0.356±0.06 respectively (p˂0.0001, t-value=4.59). However, there was no difference in the proportion of other globulin sub-fractions between cases and controls (Figures 1, 2).

**Figure 3:**
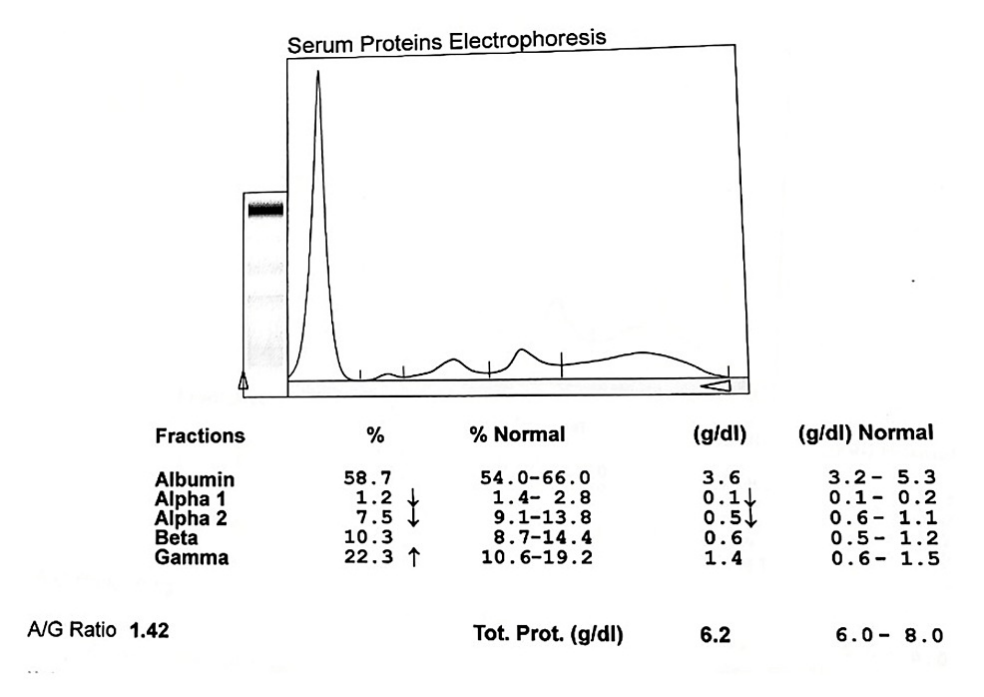
Electrophoretogram of PTB cases at two months on ATT A/G: albumin to globulin ratio, PTB: pulmonary tuberculosis, ATT: antituberculous treatment

**Figure 4:**
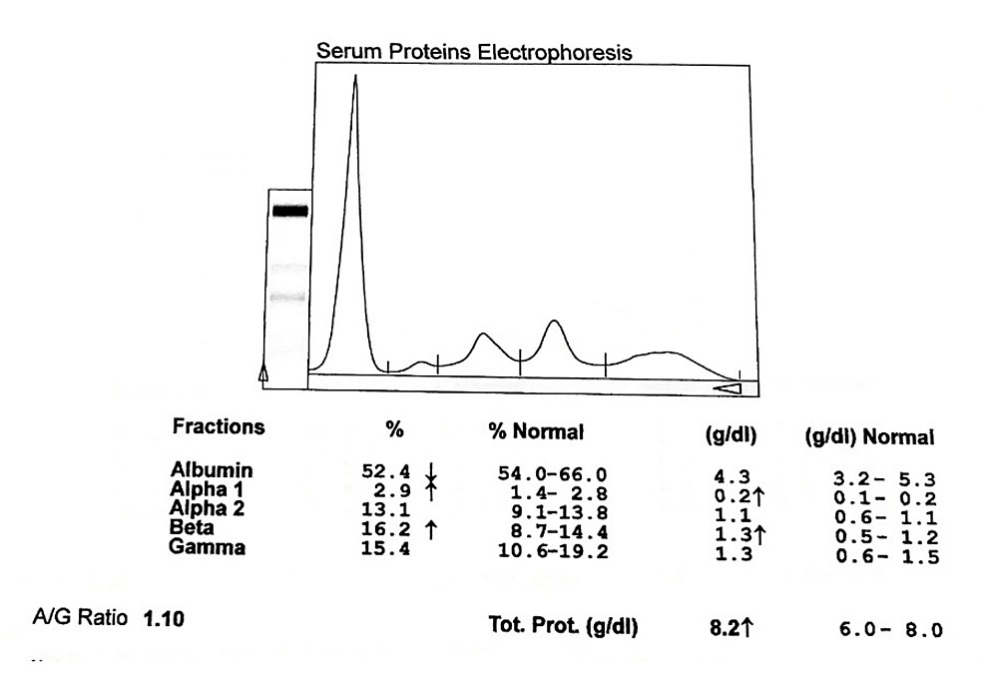
Electrophoretogram of PTB cases at four months on ATT A/G: albumin to globulin ratio, PTB: pulmonary tuberculosis, ATT: antituberculous treatment

**Figure 5:**
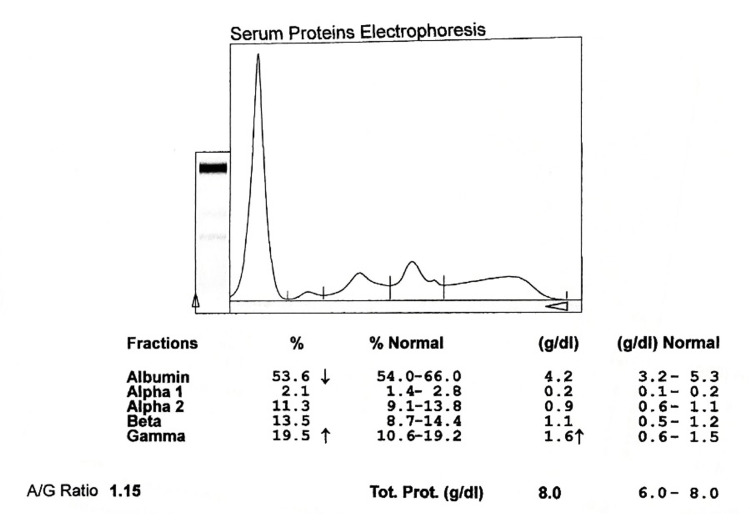
Electrophoretogram of PTB cases at six months on ATT A/G: albumin to globulin ratio, PTB: pulmonary tuberculosis, ATT: antituberculous treatment

The different fractions of globulin also show variations. The α1 globulin was stated to rise after four months of treatment and at six months level was 0.262±0.32 g/dL. The α2 globulin similarly started to rise from two months to six months from 0.773±0.35 g/dL to 0.981±0.37 g/dL. β globulin increases progressively after six months of treatment and its level is higher than in control groups. The level of γ globulin continuously decreases after anti-tuberculous treatment to 1.56±0.67 gm/dL at six months. Globulins (α1 and γ) show significant improvement in newly diagnosed and at six months of treatment with ATT (Table 2, Figures 3-5).

**Table 2:** Effect of anti-tuberculosis drug treatment on the level of different serum proteins

Duration of treatment	Total protein	Albumin	α1 globulin	α2 globulin	β globulin	γ globulin
Control	7.02±0.56	3.87±0.47	0.356±0.06	0.776±0.16	0.896±0.23	1.12±0.21
Newly diagnosed cases	6.12±0.61	1.65±0.69	0.198±0.04	0.756±0.12	0.821±0.16	2.70±0.27
2 months	6.7±0.42	2.34±0.76	0.186±0.51	0.773±0.35	0.843±021	2.56±0.12
4 months	6.86±1.03	2.96±0.54	0.262±0.32	0.881±0.16	0.887±0.26	1.87±0.98
6 months	7.43±0.56	3.64±0.34	0.282±0.23	0.981±0.37	0.967±0.18	1.56±0.67

## Discussion

Low- and middle-income countries have the highest burden of tuberculosis. Malnutrition, overcrowding, and poorly ventilated working environments are often associated with poverty and are a major risk factor for tuberculosis [14]. Although, there is an abundance of research studies on tuberculosis biochemical parameters, still much more knowledge is lagging, especially in tuberculosis infection and their disease patterns where the microbiological tests are not effective. Apart from PTB patients, other cases such as smear-negative tuberculosis, and extra-PTB are difficult to diagnose, and new biomarkers are required for serum protein parameters. Apart from serum proteins other proteins such as C-reactive protein, procalcitonin, and serum amyloid A also provide important information about the severity of tuberculosis [15].

WHO suggests preventive treatment for those who are at greater risk of reactivation of tuberculosis such as people living with HIV, children below five years of age, and patients on immunosuppressive drug therapy [16]. Some immunobiological investigations for identifying patients at risk of latent tuberculosis include positron emission tomography magnetic resonance imaging (PET-MRI), non-invasive PET-computed tomography (PET-CT), transcriptomics and other omics approaches for immunological response marker, epigenetics, and differentially expression of genes in individuals in both patients and animal models [17,18].

The clinical data analysis shows significant differences between newly diagnosed sputum-positive PTB patients and controls in levels of total protein, albumin, α, and γ globulin. According to Shingdand J (2016), the total protein and globulin were lower in tuberculosis cases as compared to controls; 6.17±1.66 and 6.76±1.66 g/dL (p=0.011) respectively. Globulin was also lower in tuberculosis cases when compared with controls at 3.22±1.90 and 3.97±0.86 g/dL (p=0.016) respectively. It may be caused by loss of appetite, malabsorption, and impaired cellular immunity [19].

Albumin is reduced during inflammatory conditions. In the present study, albumin levels were reduced in tuberculosis patients compared with controls (P<0.001) and after proper antituberculosis treatment, it started to rise gradually from intensive to continuous phase of treatment. An epidemiological study done by Cegielski JP (2012) observed that people having low levels of serum albumin were vulnerable to tuberculosis (P=0.006), whereas tuberculosis cases having low serum albumin were susceptible to death (P<0.001) [20], suggesting that albumin level may affect pathogenesis and prognosis of tuberculosis.

PTB patients had a significantly lower α globulin and higher γ globulin level as compared to the control (p˂0.0001). α1 antitrypsin and α1 acid glycoprotein are found in the α1 region of protein electrophoresis. α1 antitrypsin is a glycoprotein and it blocks proteolytic enzymes like elastase, chymotrypsin, and trypsin, and its deficiency leads to poor lung function. α1 antitrypsin concentration is higher in slow responders to ATT [21]. These bands are good potential markers of treatment response. High level of α1 acid glycoprotein has been identified as a slow response to treatment in tuberculosis patients [22].

Increased γ globulin might be due to an immune response against the invading tuberculous bacilli as reported by Damburam et al. (2012), as the difference between the percentage of gamma globulin was much higher in the cases than in the controls (mean 41.45±11.21 and 29.42±6.76%, p=0.005) respectively [23]. Arinola and Igbi reported high levels of immunoglobulin sub-fractions IgG and IgM in PTB patients [24]. Nagayama et al. also stated that hyperglobulinaemia in tuberculosis is one of the causes of pleural thickening [25]. Singanayagam (2016) found subsequent normalization of globulin in two months after anti-tuberculous treatment and suggested that measurement of globulin could be a useful marker in clinical practice for the requirement for treatment extension in tuberculosis [26].

In combination with other valuable markers of response to treatment such as weight gain [27], a decrease of signs and symptoms [28], and improvement in chest X-ray [29] with a decrease in gamma globulins level and an increase in albumin levels provides additional information to clinician about good treatment response.

## Conclusions

The cause of the decrease in total protein and albumin may be malnutrition leading to low cellular immunity. Serum protein electrophoresis should be included as routine tests in patients with PTB. It helps us to identify the prognosis of patients undergoing treatment for tuberculosis. This study is a valuable guide in deciding upon the duration of therapy necessary for drug-sensitive tuberculosis patients. This study, therefore, highlighted the complex relationship that exists between poverty and disease. Further studies on larger populations are required whether albumin and globulin could be predictors of stronger measures of success of treatment like non-relapse diseases and insignificant death related to tuberculosis.
